# Vector-borne helminths of dogs and humans – focus on central and eastern parts of Europe

**DOI:** 10.1186/1756-3305-6-38

**Published:** 2013-02-22

**Authors:** Aleksander Masny, Elżbieta Gołąb, Danuta Cielecka, Rusłan Sałamatin

**Affiliations:** 1 Department of Medical Parasitology, National Institute of Public Health–National Institute of Hygiene, 24 Chocimska, 00-791, Warszawa, Poland

**Keywords:** *Dirofilaria repens*, *Dirofilaria immitis*, *Acanthocheilonema reconditum*, Human dirofilariosis, Autochthonous

## Abstract

Comments on the article “Vector-borne helminths of dogs and humans in Europe” published in Parasites and Vectors 2013, 6:16.

## 

Dear Editor,

Vector-borne helminths (VBH) of dogs and humans are becoming recognized as an emerging problem in many parts of Europe, a situation which is reflected by the growing number of published reports from different countries. Review articles are an important means of presenting the current state of knowledge in the field. The article *“Vector-borne helminths of dogs and humans in Europe” Parasites and Vectors 2013, 6:16*[1] provides an excellent description of various aspects of VBH. However it also suffers from referring to incomplete information regarding central and eastern parts of Europe.

We would like to present data from Poland and Ukraine, omitted by the authors of the review, and correct information published in some of the cited articles. An analysis of the human cases of dirofilariosis caused by *Dirofilaria repens* detected in Poland was performed [2]. As a result, three undoubtedly autochthonous cases of human *D. repens* infection were found in the Mazovia region, i.e. in the central part of Poland till the end of 2011 [2]. In this region, canine dirofilariosis is present [3,4]. The first autochthonous human case was recorded in 2010 in Grójec (51°51'N, 20°52'E), and the next two in 2011 in Warszawa (52°35'N, 21°05'E) and Białobrzegi (51°39'N, 20°52'E). In the majority of the recognized cases (15 out of 18), the possibility of the infection taking place outside of Poland could not be excluded [2,5,6] and some of those cases were incorrectly reported as autochthonous [7]. We found a significant increase in the number of recognized human *D. repens* infections – 13 new cases in the period 2009–2011 [2].

The data on human dirofilariosis cases from Ukraine were omitted in the review [1]. We estimated that over 60%, 900 out of approximately 1500 described in the literature, European human cases of *D. repens* infection were detected in Ukraine [2,8]. These data show a shift in the endemic territory of *D. repens*. Further investigations of the epidemiological situation in central and eastern Europe could be crucial to determine the directions of the spread of dirofilariosis across the continent. Is dirofilariosis spreading from East to West or is it migrating from multiple directions towards central and northern Europe? The first autochthonous case of canine *D. repens* infection in Germany was identified in 2004, in the south-western part of the country [9]. It was suggested that the infection might spread from the south to the north of Germany, however, it was found that one of the infected dogs was brought from central Poland [10]. *D. repens* infections were confirmed to be autochthonous in dogs in central Poland, close to Warszawa in 2009 [3,4]. Therefore the question concerning the routes of spreading of *D. repens* in Europe remains open.

The first autochthonous case of canine *D. immitis* infection was detected in northern Poland (Gdynia, 54°30'N,18°32'E) in March 2012 [11]. This case indicated a new northern border of autochthonous canine *D. immitis* in continental Europe for 2012.

Moreover, our data can fill one of the blank spots on the European map of distribution of *Acanthocheilonema reconditum,* presented in the review [1]. The parasite was detected in 2011 in two dogs form central Poland during a screening of dogs for dirofilariosis [12].

## Competing interests

The authors declare that they have no competing interests.

## Authors’ contributions

All authors analyzed data on dirofilariosis in central and eastern Europe. DC and RS performed review of articles published in Ukrainian and Russian languages. AM and EG wrote the letter following the discussion with DC and RS. All authors approved the final version of the manuscript.
